# The aerotaxis of *Dictyostelium discoideum* is independent of mitochondria, nitric oxide and oxidative stress

**DOI:** 10.3389/fcell.2023.1134011

**Published:** 2023-06-15

**Authors:** Satomi Hirose, Julie Hesnard, Nasser Ghazi, Damien Roussel, Yann Voituron, Oliver Cochet-Escartin, Jean-Paul Rieu, Christophe Anjard, Kenichi Funamoto

**Affiliations:** ^1^ Graduate School of Biomedical Engineering, Tohoku University, Sendai, Japan; ^2^ Institute of Fluid Science, Tohoku University, Sendai, Japan; ^3^ Institut Lumière Matière, University of Lyon, Université Claude Bernard Lyon 1, CNRS, Villeurbanne, France; ^4^ LEHNA, UMR CNRS 5023, Université Claude Bernard Lyon 1, Villeurbanne, France

**Keywords:** *Dictyostelium discoideum*, aerotaxis, hypoxia, oxidative stress, nitrosative stress, mitochondria, microfluidics

## Abstract

Spatial and temporal variations of oxygen environments affect the behaviors of various cells and are involved in physiological and pathological events. Our previous studies with *Dictyostelium discoideum* as a model of cell motility have demonstrated that aerotaxis toward an oxygen-rich region occurs below 2% O_2_. However, while the aerotaxis of *Dictyostelium* seems to be an effective strategy to search for what is essential for survival, the mechanism underlying this phenomenon is still largely unclear. One hypothesis is that an oxygen concentration gradient generates a secondary oxidative stress gradient that would direct cell migration towards higher oxygen concentration. Such mechanism was inferred but not fully demonstrated to explain the aerotaxis of human tumor cells. Here, we investigated the role on aerotaxis of flavohemoglobins, proteins that can both act as potential oxygen sensors and modulators of nitric oxide and oxidative stress. The migratory behaviors of *Dictyostelium* cells were observed under both self-generated and imposed oxygen gradients. Furthermore, their changes by chemicals generating or preventing oxidative stress were tested. The trajectories of the cells were then analyzed through time-lapse phase-contrast microscopic images. The results indicate that both oxidative and nitrosative stresses are not involved in the aerotaxis of *Dictyostelium* but cause cytotoxic effects that are enhanced upon hypoxia.

## 1 Introduction

Oxygen is vital for many living organisms (Hsia et al., 2013). The oxygen concentration inside multicellular organisms is lower than that in the atmosphere due to oxygen consumption by cells, and changes spatially and temporally (Vaupel et al., 1991). The variations of oxygen environment affect various cell behaviors physiologically and pathologically (Semenza, 2000; Eltzschig and Carmeliet, 2011). Aerotaxis, the motion of cells toward optimal oxygen levels, is well known in bacteria (Taylor et al., 1999), but the characteristics and mechanisms of aerotaxis in eukaryotic cells are poorly elucidated. Although intracellular protein hypoxia-inducible factors (HIF) generally express their functions by transcriptionally activating genes in the nuclei (Semenza, 2007), they do not seem to induce a rapid directional motility change. Recently, an *in vitro* study reported a clear oxygen-directed migration of both epithelial and breast tumor cells under a self-generated hypoxic environment caused by cellular oxygen consumption in a confined environment (Deygas et al., 2018; Mikaelian et al., 2022). Such oxygen-directed migration was also observed even after the knockout of HIF in the cells, suggesting a HIF-independent aerotaxis. An oxidative gradient seems to overlap the oxygen gradient and is required to direct aerotactic motility through activation of the epithelial growth factor receptor (EGFR). The cysteine 797 located near the ATP binding site of EGFR was shown to be a target of redox regulation (Truong et al., 2016) and might be the target of the hypoxia induced oxidation that potentiate its tyrosine kinase activity. Most of the dioxygen is metabolized in mitochondria to produce ATP as part of respiration processes (Spinelli & Haigis, 2018). Mitochondria generate a burst of oxidant molecules, both reactive oxygen species (ROS) and reactive nitrogen species (RNS) during hypoxia (Castello et al., 2006; Poyton et al., 2009; Doorly and Graciet, 2021), and inactivation of mitochondrial respiration prevents aerotaxis (Mikaelian et al., 2022). It was thus hypothesized that in hypoxic condition an oxygen gradient will cause cells to generate a secondary redox gradient that would modulate EGFR activity to direct cell migration toward higher oxygen concentrations (Mikaelian et al., 2022).

*Dictyostelium discoideum* vegetative amoeba also shows collective migration toward an oxygen-rich region. In a confined spot assay, *Dictyostelium* cells exhibited long-lasting and highly stable migration towards oxygen-rich regions (i.e., aerotaxis) guided by a self-generated oxygen gradient, forming a stable ring of cells to escape from the hypoxic region (Biondo et al., 2021; Cochet-Escartin et al., 2021). Moreover, the aerotaxis of *Dictyostelium* was successfully confirmed under controlled oxygen gradient generated by microfluidic devices with gas channels for supply of gas mixture of nitrogen and oxygen (Hirose et al., 2022). Under hypoxic conditions, below 2% O_2_, which corresponds to 25 µM O_2_ in the culture medium, the migration of *Dictyostelium* cells was enhanced (aerokinesis), and the oxygen gradient guided the cells toward the oxygen-rich region (aerotaxis). A catalase-deficient strain of *Dictyostelium* demonstrated an aerotactic ring formation quicker than the wild type, suggesting that hydrogen peroxide (H_2_O_2_) could play a role in the aerotaxis of *Dictyostelium* (Biondo et al., 2021). How cells sense an oxygen gradient and integrate it to direct motility is not clear as this phenomenon is little studied contrary to response to hypoxia. It is possible that aerotaxis uses some of the hypoxia sensing mechanisms. In eukaryotes, oxygen is mainly used to generate energy through oxidative phosphorylation occurring in mitochondria. Hypoxia can thus be detected indirectly through the lack of energy and increase in free radicals (ROS and RNS) resulting from impaired mitochondrial functions (Castello et al., 2006; Hernansanz-Agustín et al., 2017). A more direct way to detect hypoxia involves oxygen-dependent enzymes that will regulate downstream responses. The only well-documented oxygen sensing system in *Dictyostelium* is PhyA, a member of the oxygen-dependent prolyl-hydroxylase domain (PHD) proteins also found in mammals and plants (West et al., 2007; Zhang et al., 2012). PhyA is a prolyl hydroxylase that acts as an oxygen sensor to control the terminal phase of *Dictyostelium* multicellular development that results into the differentiation of spore and stalk cells. Since PhyA present a low affinity of O_2_, its activity is restricted under 21% O_2_. In presence of normal level of oxygen, PhyA hydroxylates a proline on Skp1, an adaptor subunit of the Skp1–Cul1–F-box (SCF) class of E3 ubiquitin triggering its glycosylation. The modified Skp1 will in turn associate with its targets to trigger their polyubiquitination and degradation by the proteosome (West & Blader, 2015). PhyA activity is reduced under 10% O_2_, resulting into the accumulation of unmodified Skp1, preventing terminal differentiation from proceeding. PhyA is expressed during *Dictyostelium* multicellular development and control the culmination phase, such that it happens in a place with normal oxygen level such as the soil surface rather than underground (West et al., 2007).

Other candidates include flavohemoglobins, oxygen-dependent enzymes that neutralize nitric oxide (NO), a source of RNS that also acts as a signaling molecule regulating the hypoxic response in many organisms (Castello et al., 2006). Flavohemoglobins are found in many bacteria, fungi and protists where it oxidizes NO into nitrate (Cánovas et al., 2016) to maintain NO homeostasis. While NO is produced at low level as part of nitrogen metabolism, high level can occur during hypoxia due to dysfunction of mitochondrial cytochrome c oxidase (Tischner et al., 2004; Gupta et al., 2005; Planchet et al., 2005; Castello et al., 2006) but can also results from fermentation of nitrite in acidic pH or be released by plants or animals as part of antibacterial defense. The oxidation of NO by flavohemoglobin is very efficient in saturation oxygen concentration but the reaction can also occur in hypoxic condition when oxygen is in micromolar range (Poole et al., 1996). In anaerobic condition, flavohemoglobins appear able to detoxify NO at a slow rate through an alternate reaction (Kim et al., 1999). As such, flavohemoglobins were shown to protect organisms from NO toxic effects both in high and low oxygen conditions (Forrester & Foster, 2012). *Dictyostelium* genome encodes two flavohemoglobins genes, *fhbA* and *fhbB*, that were successfully inactivated (Iijima et al., 2000). *Dictyostelium* flavohemoglobin proteins share well conserved amino-acid sequences and structure with the characterized bacterial and fungal homologs shown to act as NO dioxygenases (Dröge et al., 2014). While the enzymatic activity of FhbA and FhbB has not been tested, the double null mutant *fhbA* ^*-*^ *B* ^*-*^ displays poor viability when challenged with NO generating chemicals, consistent with their expected NO dioxygenases functions (Iijima et al., 2000). The intracellular localization of *Dictyostelium* flavohemoglobins has been not investigated but in organisms that also possess two flavohemoglobin genes, such as filamentous fungi, one isomer is at least partly located in the mitochondrial matrix despite lacking a classical targeting sequence while the other isomer is mostly cytoplasmic (Forrester & Foster, 2012). In yeast, the YHB1 flavohemoglobin is distributed between the cytosol and mitochondrial matrix in normoxia but relocates fully to mitochondria in anaerobic condition (Cassanova et al., 2005). *Dictyostelium* cells generate NO at the onset of multicellular development to regulate the initiation of cAMP pulses (Tao et al., 1997). This NO generation coincides with the strong downregulation of *fhbA* and *fhbB* expression observed by RNA-seq (Rosengarten et al., 2015). *fhbA* and *fhbB* are re-expressed at low level when aggregates are formed. cAMP is secreted by *Dictyostelium* to act as a chemoattractant that directs cell motility during the aggregation phase (Pal et al., 2019). It is thus possible that flavohemoglobins act as oxygen sensor that would regulate NO level produced during hypoxia. NO could then act as a secondary signal to direct cellular motility upward oxygen gradients.

In this context, available mutants in genes encoding for potential oxygen sensors or signaling intermediates were tested for their ability to form aerotactic rings of cells when a micro-colony (spot of cells) was covered with a glass coverslip. Out of the mutants tested, including members of the PhyA-Skp1 pathway, only one displayed an abnormal phenotype during the spot assay. A null mutant of the flavohemoglobin gene *fhbB* presented a poorly defined ring that expanded at a slower rate than wild-type controls. Therefore, we investigated the role of flavohemoglobins and the effects of oxidative stress on aerotaxis. The migratory behaviors of wild type and mutant *Dictyostelium* cells were observed both with the spot assay and with microfluidic devices generating an oxygen gradient. Chemicals were used to inhibit mitochondria functions, source of free radical or anti-oxidant. The trajectories of the cells were then analyzed with time-lapse phase-contrast microscopic images to draw the aerokinetic and aerotactic features of *Dictyostelium* cells under these pharmacogenetic treatments. Thanks to complementary cell respiration and proliferation experiments, micro-colony experiments were modeled with a Go-or-Grow mean field model previously introduced (Cochet-Escartin et al., 2021) in order to confirm the existence of a true aerotactic response in all analyzed experiments. This complementary experimental and theoretical approach indicates that neither flavohemoglobins nor oxidative stress are involved in the aerotaxis of *Dictyostelium* contrary to expectations. Instead, our data indicates that hypoxia increases the toxic effects of oxidative stress on cell motility, leading indirectly to an impaired aerotactic motility. Surprisingly, we observed that for *Dictyostelium*, mitochondrial respiration is dispensable both for generation of an oxygen gradient and for aerotactic response.

## 2 Materials and methods

### 2.1 Cells and strains

The wild types AX2 and AX3, and the flavohemoglobin null mutants *fhbA* ^-^, *fhbB* ^-^ and *fhbA* ^-^ *B* ^*-*^ strains of *Dictyostelium* cells were provided by the National BioResource Project (NBRP Nenkin, Japan) and cultured axenically with HL5 medium (HLF2; Formedium, United Kingdom) containing 100 mM glucose and 1/1,000 penicillin-streptomycin (P/S) (15140-122; Gibco, United States) on cell culture dishes at 22°C or under agitation. PhyA-strain is a kind gift from C. West (University of Georgia, United States).

### 2.2 Chemicals and reagents

Sodium nitroprusside dihydrate (SNP) (71778; Sigma-Aldrich, United States), which releases NO, was added at 10–100 µM to the culture medium to investigate the effect of RNS on the aerotaxis. H_2_O_2_ (18084-00; Kanto Chemical, Japan) was added at 100–500 µM to investigate the effect of ROS on the aerotaxis. Ethidium bromide (EtBr) (E1510, Sigma-Aldrich), which inhibits mitochondria DNA replication, was added to the culture medium 24 h before experiments at 30 μg/mL for pretreatment of the cells. Oligomycin (O4876, Sigma-Aldrich) was used at 10 μg/mL, and antimycin A (A8674, Sigma-Aldrich) was used at 37.5 µM. Tiron (4,5 dihydroxy-1,3-benzene-disulphonic acid; D7389, Sigma-Aldrich) was used at 10 mM.

### 2.3 Microfluidic devices

A double-layer microfluidic device with oxygen-controllability was used (Supplementary Figure S1) (Hirose et al., 2022). The device was 30 × 30 mm square, and the thickness was 4 mm. Inside the device, two gas channels in a 1-mm interval were located at 0.5 mm from the bottom surface, perpendicularly crossing above three parallel media channels on the bottom (Supplementary Figure S1A). The width and height were 2 mm and 0.15 mm for all channels, respectively. The device mainly consisted of polydimethylsiloxane (PDMS, Sylgard 184 Silicone Elastomer Kit, The Dow Chemical Company, United States) and contained a polycarbonate film to prevent gas infusion from the atmosphere. The *x* and *y*-axes were defined to be parallel and perpendicular to the media channels, respectively, and the *z*-axis was in the vertical direction. The origin was set at the center of the device. The oxygen concentration in the device was controlled by gas exchange between the channels, supplying gas mixtures at predefined oxygen concentrations to the gas channels. The oxygen concentration in the device was computed by three-dimensional numerical simulation using commercial multiphysics software (COMSOL Multiphysics ver. 5.5, COMSOL AB, Sweden) and verified using an oxygen-sensing film which emitted phosphorescence quenched by oxygen (Hirose et al., 2021). The computational and experimental results showed almost the same tendency especially for the minimum value of oxygen concentration, showing an oxygen concentration gradient from 0.4% to 21% O_2_ was generated along each media channel in the *x*-direction when the gas mixtures containing 0% and 21% O_2_ were supplied to the left and right gas channels, respectively (gray solid line in Supplementary Figures S1B–F). At the most hypoxic location at *x* = −1.6 mm, the slope of the oxygen concentration gradient inverted.

*Dictyostelium* cells were harvested from the culture dishes and introduced into the media channels in the microfluidic device at 2 × 10^6^ cells/mL (400 cells/mm^2^) with the HL5 culture medium. After incubation for 20 min to adhere *Dictyostelium* cells to the bottom surface of the media channels, the HL5 culture medium was replaced by fresh one. Here, depending on the experimental conditions, reagents were added to the HL5 culture medium. For accurate comparisons, each device was set to have channels for control condition and each of another two conditions at the same time. The device was placed in a stage incubator (TP-CHSQ-C, Tokai Hit, Japan) mounted on an inverted microscope (IX83, Olympus, Japan), and controlled at 22°C. Just after gas mixtures of oxygen and nitrogen were supplied at 30 mL/min to the left and right gas channels, time-lapse imaging was started.

A second type of microfluidic device, with a Y-shape channel was used to generate a chemical gradient of soluble molecules (Figure 3A). The device was placed in the same stage incubator mounted on the microscope, and controlled at 22°C. The *x* and *y*-axes were defined to be perpendicular and parallel to the merged channel, respectively. The origin was set at the junction of the channels from the two inlets. *Dictyostelium* cells were first allowed to adhere on the microchannel in HL5 culture medium for 20 min. HL5 culture media without and with a pharmacological substance at a concentration *C*_0_ (SNP at 100 µM or H_2_O_2_ at 500 µM) were injected from the left and right inlets, respectively, at 1 mL/h, and time-lapse imaging was then started. The average velocity was *U*_*0*_ = 0.37 mm/s in the merged channel inducing a wall-shear stress around 4.4 mPa low enough not to affect cell migration and adhesion on the bottom glass (Rupprecht et al., 2012). The profile of chemical concentration *C* (*x*, *y*) at a given position (*x*, *y*) was calculated by the following equation:

$$C\left(x,y\right)=\frac{C_{0}}{2}\left[1+\mathrm{erf}\left(\frac{x}{2}\sqrt{\frac{U_{0}}{Dy}}\right)\right]$$ (1)

where *D* is the diffusion coefficient of the chemical substance in water. For the H_2_O_2_ gradient we used *D* = 1.4 × 10^−3^ mm^2^/s (van Stroe-Biezen et al., 1993). Addition of 100 µM SNP leads to rapid increase of NO in the media to reach a prolonged plateau after 25 min (Bradley & Steinert, 2015). As the experimental set-up require 30 min, we expect a constant level of NO for the duration of the assay, hence for the SNP gradient experiment. We used the diffusion constant of NO, *D* = 2.2 × 10^−3^ mm^2^/s (Zacharia & Deen, 2005), to compute the relative NO concentration profile. Cells were imaged and tracked between 1 h and 2 h after starting the media flow.

### 2.4 Imaging and analyzing cell migration in microfluidic devices

Phase-contrast microscopic images of the central part of each media channel including the region of interest (ROI) displayed in Supplementary Figure S1A and were taken by the microscope with a ×4 objective lens (UPLFLN4XPH, Olympus) every 30 s for 2 h. The positions of *Dictyostelium* cells were detected at each time step by analyzing the images using ImageJ (National Institutes of Health, United States) with its built-in plugin, Find Maxima. Then, the individual cells were tracked using MATLAB (MathWorks, United States) by minimizing the total of squared displacements with a published tracking code (Blair and Dufresne, 2008). All trajectory data were analyzed using in-house MATLAB programs. The migration distance in 1 min between the time steps *t*_*i*_ and *t*_*i*+1_ was defined as *dl*_*i*_, which was decomposed into the *x* and *y*-directional displacements *dl*_*x,i*_ and *dl*_*y,i*_, respectively (*dl*_*i*_^2^ = *dl*_*x,i*_^2^ + *dl*_*y,i*_^2^), where the *x*-direction is defined as the gradient direction for both devices. The average speed *v =* $\bar{{dl}_{i}}$ and the average *x*-directional velocity *v*_*x*_ *=* $\bar{{dl}_{x,i}}$ were averaged from all 1-min cell displacements in Δ*x* = 200 µm wide slabs as a function of *x*-position. The aerotactic index was defined as *AI =* $\bar{{dl}_{x,i}/{dl}_{i}}$ referring to a chemotactic index normally used as a metric for chemotaxis (Amselem et al., 2012). The maximal *AI*_max_ was measured in each individual experiment as the maximum value of *AI* on the positive side (*x* > −1.6 mm) since the oxygen gradient was better controlled than on the negative side. A chemotaxis index *CI =* $\bar{{dl}_{x,i}/{dl}_{i}}$ was computed to analyze the data by experiments with the Y-shaped microfluidic devices.

### 2.5 Spot assay and ring speed kymographs

The confined spot assay offers a quick and robust way to test whether cells can generate oxygen gradients and move aerotactically (Deygas et al., 2018; Biondo et al., 2021; Cochet-Escartin et al., 2021). A drop of either 2, 3 or 5 µL of a cell suspension containing 2,000, 3,000 or 5,000 cells respectively was carefully deposited on a dish. The drop was incubated for 30 min in humid atmosphere at 22°C before adding 2 mL of HL5 culture medium. An 18 mm or 22 mm diameter round glass coverslip cleaned with ethanol then thoroughly rinsed in HL5 was gently deposited on top of it. Image acquisition was performed using a lens-free imaging device (Cytonote 6W, Iprasense, France) or the aforementioned inverted microscope. Images were taken every 5–15 min for 24 h. The software Horus (Iprasense, France) was used to process and reconstruct the Cytonote images, and a homemade ImageJ macro based on the polar transformer plug-in was used to construct kymographs from the original images by polar transformation and *z*-stacking of frames. The ring speed was determined by calculating the slope of the front density band associated with ring propagation on these kymographs between 16 h and 24 h. To improve signal to noise ratio in kymographs, images were filtered from their background by using the Find-Edges function of ImageJ before applying the polar transformation.

### 2.6 Oxygen consumption and proliferation experiments

For consumption measurements of *fhb* null mutant and for those of the respiratory inhibitors oligomycin and antimycin A (Supplementary Figure S6C), about 10^6^ total cells suspended in HL5 culture medium were placed in a respirometer (Oxygraphs 2k high-resolution respirometers, Oroboros Instruments, Austria) to determine oxygen concentration changes over time. After the measurement of the basal respiration, oligomycin (10 μg/mL) or antimycin A (37.5 µM) were added successively. Oxygen consumption were calculated using the change of oxygen level over time and normalized per cell. For consumption measurements of EtBr treated cells or in presence of SNP or H_2_O_2_ (Supplementary Figure S6D), a 6-mL glass vial was filled with 2 × 10^7^ cells suspended in HL5, and closed it with a cap through which an optical oxygen probe was plunged. The cap was carefully sealed around the probe to avoid external oxygen entering the bottle. We measured the change of oxygen level over time with this probe coupled to its oxymeter (OXROB3 robust probe and Firesting oxymeter, Pyroscience, Aachen, Germany).

For proliferation experiments, cells were plated at about 10^4^ cells/cm^2^ in 6-well plates, and imaged and counted with the lens-free imaging device or the inverted microscope every 30 min for 24 h.

### 2.7 RT-qPCR

Four plates containing 2.3 × 10^6^ A×3 cells in 1.5 mL HL5 culture medium were prepared with either no chemicals, 100 µM SNP or 100 µM H_2_O_2_. The two plates containing SNP or H_2_O_2_ and a plate without chemical were placed at 22°C and 21% O_2_. The last plate containing cells in HL5 culture medium was incubated at 22°C and 0.4% O_2_. After 3 h, total RNA was extracted (RNeasy Plus Mini Kit, 74134, Qiagen, Germany), quantified and adjusted at 1 µg to synthesise cDNA (First Strand cDNA Synthesis Kit for RT-PCR, 11483188001, Roche, Switzerland). Then a qPCR was performed (DyNAmo Flash SYBR Green qPCR Kit, F415L, Thermo Fisher Scientific, United States) using the following primers: *rnlA*-F (5′-GCA​CCT​CGA​TGT​CGG​CTT​AA-3′), *rnlA*-R (5′-CAC​CCC​AAC​CCT​TGG​AAA​CT-3′), *cxgE*-F (5′-ATG​TCC​CAC​GCA​TTA​CCA​G-3′), *cxgE*-R (5′-CAT​AGG​CAG​CGT​AAA​AAT​CTT​CTC-3′), *cxgS*-F (5′-AAG​TTG​TTA​AAT​CTC​AAC​TC-3′), *cxgS*-R (5′-TTT​ATC​AAC​GCC​ATA​TTT​AA-3′), *fhbA*-F (5′-GAA​CTC​ACA​TCA​GAC​ATT​AC-3′), *fhbA*-R (5′-CTA​CAT​AGT​CAC​CAG​CTG​GA-3′), *fhbB*-F (5′-GAT​GGT​AAA​GAG​ATA​GCT​AC-3′), and *fhbB*-R (5′-CTG​ATA​AAC​TGT​AAT​GTC​TGA​C-3′). The experiments were performed in duplicate for each gene. The relative gene expression was determined using the 2^−ΔΔCt^ method (Livak & Schmittgen, 2001) using *rnlA* as a housekeeping gene.

### 2.8 Statistical test

For each measurement type, experiments were repeated three to six times. Significant differences between three or more conditions were evaluated by one-way ANOVA or two-way ANOVA followed by a post-Tukey test for multiple comparisons. In each test, statistical significance and the tendency toward significance were inferred at *p < 0.05 and †*p* < 0.1, respectively.

### 2.9 Mean-field “Go-or-Grow” model

The simulations were performed using a numerical mean-field “Go-or-Grow” model coded in the Python language, which we recently introduced to explain ring propagation in confined spot experiments (Cochet-Escartin et al., 2021). The mean field model used for the aerotactic dispersion consisted of coupled reaction-diffusion equations for both oxygen and cell concentrations respectively:

$$\frac{\partial C}{\partial t}=D_{O_{2}}\Delta C-b_{0}\left(C\right)\rho$$ (2)

$$\frac{\partial \rho }{\partial t}=D_{cells}\;\Delta \rho +r_{o}\left(C\right)\rho -\vec{\nabla .}{\vec{J}}_{aero}$$ (3)

where *C* is the oxygen concentration, *t* is the time, $D_{O_{2}}$ is the oxygen diffusion constant, *b*_0_ is the cell oxygen consumption, *ρ* is the cell density, *D*_cells_ is the cellular diffusion constant, *r*_0_ is the cell proliferation rate, and ${\vec{J}}_{aero}$ is the advection term. From our recent observations that the aerotactic displacements are more correlated to the relative oxygen gradient $\left(\frac{\vec{\nabla }C}{C}\right)$ than to the absolute gradient $\vec{\nabla }C$ (Hirose et al., 2022), we propose a slight modification to the advection term in the shape of ${\vec{J}}_{aero}=\rho {\vec{v}}_{aero}$ , with:

v→aero=χ l∇→CC)11+eC−C0s (4)

χ is a proportionality constant called aerotactic bias (unique for each experimental condition, see below), l = 10 µm is the approximate cell size, and the latter two terms comprise the response function to oxygen relative gradient. We choose an initial cell distribution and a boundary condition for oxygen *C* (*L =* 9 mm) = 21% O_2_ mimicking the experimental situation. Mesh sizes of Δ*x* = 10 µm and Δ*t* = 0.05 min were used. To avoid negative O_2_ values a threshold *C*_1_ = 0.5% O_2_ was chosen below which cells consume oxygen relative to the local ambient oxygen concentrations. We also used a proliferation threshold below which cells stop dividing *C*_2_ = 0.2% O_2_ as recently measured (Carrère et al., 2023). This is also a slight modification to our initial Go-or-Grow model for which *C*_0_ = *C*_2_ (Cochet-Escartin et al., 2021).

The aerotactic bias, the oxygen threshold, and the steepness factor in Eq. 4 were respectively updated to *χ*_0_ = 30 μm/min, *C*_0_ = 0.4% O_2_ and *s* = 0.1% O_2_ upon further analysis of density accumulation of cells and maximum aerotactic displacement along the gradient perceived in the microfluidic device experiments with the AX2 in HL5 control condition (Ghazi et al., in preparation). For other conditions, *C*_0_ and *s* were kept constant as we never observed any shift in the range of oxygen concentrations triggering an aerotactic response, but *χ* was set proportional to the measured aerotactic *x*-directional velocity *v*_*x*_ (Supplementary Figure S1C; Supplementary Table S1), as $\chi =\chi_{0}\frac{v_{x}}{v_{x0}}$ where *v*_*x*0_ is the aerotactic speed of the control condition reported in Figures 2E, H. The cellular diffusion constant *D*_cells_ plays a minor role on the ring propagation speed or ring formation time (Ghazi et al., in preparation). It was not systematically measured but we used a scaling relation motivated by former studies (Golé et al., 2011), $\sqrt{\frac{D_{cells}}{D_{0}}}=\frac{v}{v_{0}}$ where *D*_0_ = 30 μm^2^/min (Cochet-Escartin et al., 2021) and *v*_0_ = 5 μm/min (Supplementary Figure S3D) are the diffusion constant and average speed of the AX2 control condition in the ring region. All parameter values, cell oxygen consumption *b*_0_, aerotactic *x*-directional velocity *v*_*x*_, absolute displacement speed *v*, cell proliferation rate *r*_0_, were taken directly from experimental observations discussed in this article and summarized in Supplementary Table S1. Oxygen diffusion constant in HL5 medium at 22°C was taken from the one in pure water at 20°C: $D_{O_{2}}$ = 2,000 μm^2^/s (Cochet-Escartin et al., 2021). On one hand, salt and sugar present in HL5 decrease $D_{O_{2}}$ by about 100 μm^2^/s, but on the other hand, temperature increases $D_{O_{2}}$ by about 50 μm^2^/s/°C (Goldstick et al., 1976; Jamnongwong et al., 2010).

## 3 Results

### 3.1 Abnormal motility of flavohemoglobin B null mutant upon hypoxia

**FIGURE 1:**
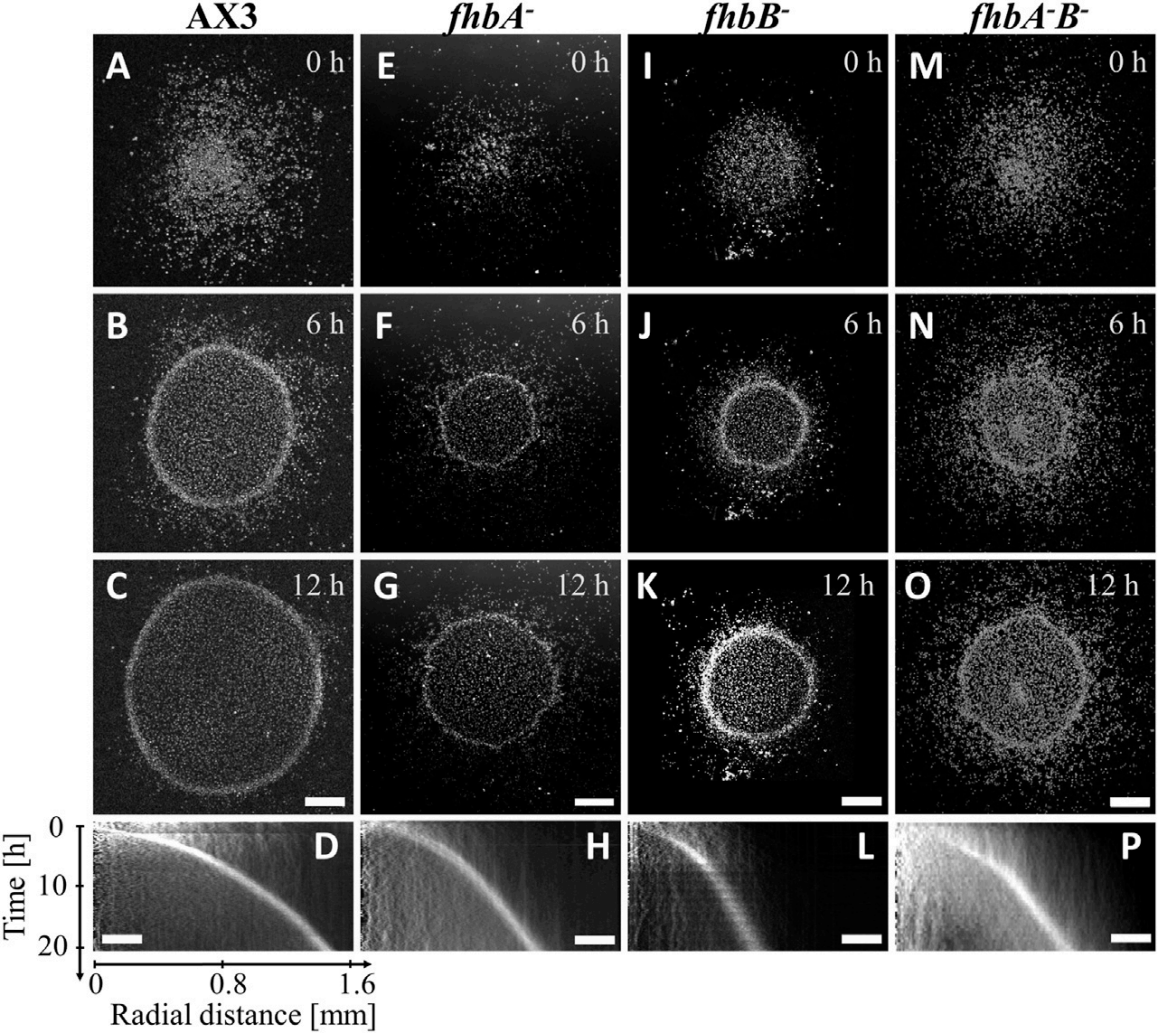
*fhb* KO cells migrate toward oxygen in the confined spot assay. Snapshots **(A,E,I,M)** right after, **(B,F,J,N)** 6 h after and **(C,G,K,O)** 12 h after covering the colony with a glass coverslip non permeable to O_2_: cells quickly consume available O_2_ and move outwardly with a characteristic ring front. **(D,H,L,P)** Kymographs of mean image intensity (horizontal axis: radial distance from spot center; vertical axis: time). **(A–D)** AX3 parent cell line, **(E–H)** *fhbA* ^-^, **(I–L)** *fhbB* ^-^, **(M–P)** *fhbA* ^-^B^-^. Bars: 500 μm and 250 µm for snapshots and kymographs (horizontal axis) respectively.

Micro-colonies of various strains were confined with a glass coverslip and observed by videomicroscopy. The behavior of the PhyA null mutant or of the one of the wild type AX3 cells were similar to the one previously described with AX2 cells (Figures 1A–D; Supplementary Figure S2). After less than an hour, the oxygen depletion at the center of the colony induced an increase of aerokinetic motility and the formation of an expanding ring of aerotactic cells. This resulted in a sharp density profile, the ring being two to three times of higher density than the interior of the colony. All of *fhb* null mutants presented a much slower and rougher ring of cells than the AX3 strain (Figure 1), the slowing down of the ring being especially more marked and significant for the *fhbB* ^-^ and *fhbA* ^-^B^-^ (Supplementary Figure S3C). Furthermore, small aggregates of cells were frequently observed inside the mutant colonies while they were very rare for the wild type.

In order to test solely the aerotaxis of *fhb* null mutants in imposed oxygen gradients, and not the intermingled response to self-generated gradients with oxygen consumption and cell proliferation, we used a double-layer microfluidic device generating a 0.4%–21% O_2_ gradient (Supplementary Figure S1A). In such a device, at the most hypoxic location where the slope of the oxygen concentration gradient was inverted, the directionality of the cell migration was changed from the -*x* direction for *x* < −1.6 mm to +*x* direction for *x* > −1.6 mm, implying migration toward higher oxygen levels [Supplementary Figure S1B with the already reported AX2 strain (Hirose et al., 2022)]. The existence of such an inversion even when the signal-to-noise in cell displacement is low is a signature of an aerotactic response. Cells migrated also in the *y*-direction but the average of the *y*-directional displacements were almost zero regardless of the position (Supplementary Figure S1B). The AX2 strain migrated faster in the hypoxic region than in the normoxic region (positive aerokinesis, Supplementary Figure S1C).

**FIGURE 2:**
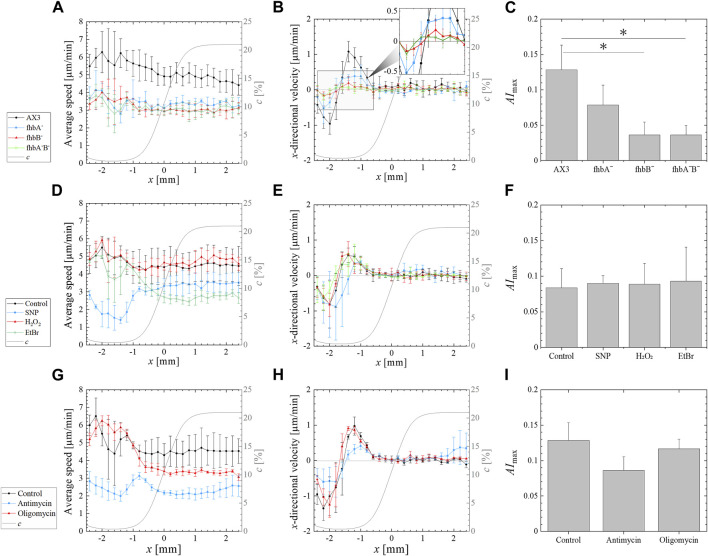
Effect of various treatments on the aerotactic response of *Dictyostelium* to an imposed 0.4%–21% external oxygen gradient (left to right) generated by supplying pure nitrogen and air to the gas channels of a double-layer microfluidic device. **(A,D,G)** Average speed (i.e., absolute value of the displacement per minute) vs. the position *x* along the gradient direction. **(B,E,F)** *x*-directional velocity (i.e., displacement along gradient direction per minute) vs*.* the position *x* along the gradient direction. The computed profile of oxygen concentration *c* is overlapped (gray solid line) on these profiles showing the oxygen gradient from 0.4% O_2_ to 21% O_2_ (right axis). **(C,F,I)** Maximal (positive) value *AI*_max_ of the average aerotactic index. The values and error bars on profiles and on *AI*_max_ are means and standard deviations over independent experiments. Significant differences were assessed by one-way ANOVA followed by Tukey’s *post hoc* test for multiple comparisons. *p < 0.05. The various experimental conditions are the following. **(A–C)** Response of *fhb* null mutants and associated AX3 parent cell line (*N* = 3 for *fhbA* ^*-*^ and *fhbA* ^*-*^ *B* ^*-*^, *N* = 6 for *fhbB* ^*-*^ and AX3). **(D–F)** Effect of SNP, H_2_O_2_ and EtBr on the AX2 control strain (*N* = 3 for SNP, H_2_O_2_ and EtBr treated cells, *N* = 9 for the control AX2). **(G–I)** Effect of oligomycin or antimycin treatment on the migration of the control AX2 cells (*N* = 3 for all conditions).

The parental AX3 strain presented a higher non-directional motility than the mutants in all oxygen concentrations (Figure 2A) and displayed a strong aerotactic bias when the oxygen level was under 1.5% O_2_ (Figure 2B), resulting in a maximal aerotactic index *AI*_max_ of 0.13 (Figure 2C). The average speed of all *fhb* null mutants was significantly reduced (Figure 2A), but all exhibited an aerotactic displacement. Even if for the *fhbB* ^-^ and *fhbA* ^*-*^ *B* ^*-*^ strains, it was just measurable (see inset of Figure 2B). As a result, the *AI*_max_ was slightly reduced in *fhbA* ^-^ strain comparatively to wild type, but reduced by more than half for the *fhbB* ^-^ and *fhbA* ^*-*^ *B* ^*-*^ strains, indicating a strong reduction in the ability of the cells to properly sense or respond to oxygen gradients in these mutants. We performed a comparative random motility assay in plastic dishes at 21% and 0.4% O_2_ (Supplementary Figure S4). The AX3 strain exhibited a higher motility in the hypoxic condition confirming the results of Figure 2A and the flavohemoglobin mutants motility was normal in normoxia but slightly reduced by hypoxia (Supplementary Figure S4).

In many organisms, flavohemoglobins expression is induced by NO, while induction upon hypoxia is uncommon (reviewed in Forrester and Foster, 2012). Therefore, the level of expression of *fhbA* and *fhbB* genes in wild type cells were tested by RT-qPCR in response to hypoxia and SNP, a chemical source of NO or H_2_O_2_ as a generic source of ROS (Table 1). Both flavohemoglobins expression were strongly induced after 3 h in 0.4% O_2_. Strong induction was also observed for *cxgS,* an optional subunit of cytochrome c oxidase induced by hypoxia, used here as positive control (Bisson et al., 1997). As expected, *cxgE*, the alternate optional subunit of cytochrome c oxidase expressed only during growth in normoxia was sharply downregulated at 0.4% O_2_ (Bisson et al., 1997). On the other hand, treatment with SNP, a classical source of NO, did not induce *fhbB* expression and rather slightly repressed *fhbA*. As mitochondria also release ROS, H_2_O_2_ effect was tested but did not affect the flavohemoglobins expression. Thus, low oxygen concentration appears to be key to induce the expression of flavohemoglobin genes, reinforcing the possibility that the proteins are important in this condition required for aerotaxis to appear.

**TABLE 1:** Changes in expression level of a series of genes in response to oxidative stress or hypoxia. Wild type cells in petri dishes were incubated for 3 h with 100 µM SNP as source of NO, 100 µM H_2_O_2_ or in a hypoxia chamber with 100% nitrogen, resulting in a final concentration of 0.4% O_2_. RNA was extracted and gene expression quantified by RT-qPCR using untreated cells as standard.

	Conditions		
Gene	H_2_O_2_	SNP	Hypoxia
*cxgE*	0.95	1.09	0.10
*cxgS*	0.77	0.64	3.14
*fhbA*	0.84	0.44	6.02
*fhbB*	1.13	0.93	4.07

### 3.2 Oxidative stress does not affect aerotaxis

Micro-colonies of wild type AX2 cells were confined in HL5 supplemented with either H_2_O_2_ as a source of ROS or with SNP as a source of RNS by generating NO in the media. Neither product prevented the formation of the expanding ring of cells even though the ring speed was reduced with SNP or H_2_O_2_ (Supplementary Figures S3A, S5). It was not possible to use much higher concentrations of these chemicals as they prevented cell adhesions or killed the cells at 1 mM. As an alternate way to change the potential effect of ROS, we used Tiron, a free radical scavenger that had previously been used to prevent oxidative stress in *Dictyostelium* (Bloomfield & Pears, 2003). Addition of 10 mM Tiron to confined wild type cell had no visible effect on ring formation appearance or migration speed (Supplementary Figure S5).

To detect eventual fine effect of free radical, SNP and H_2_O_2_ were applied to wild type cells placed in the microfluidic device previously described. However, lower concentration had to be used as they impair cell adhesion to the glass bottom of the device. The 100 µM H_2_O_2_ did not affect migration speed or response to oxygen gradient of the cells (Figures 2D–F). On the other hand, the 100 µM SNP reduced only slightly the cell speed at 21% O_2_ (3.5 μm/min versus 4.5 μm/min) but dramatically at 0.4% O_2_ (1.5 μm/min versus 5 μm/min) (Supplementary Figure S3D). Despite this strong speed reduction, the SNP-treated cells still responded to oxygen gradients and the maximum aerotactic index remained similar to that of untreated cells (Figure 2F).

**FIGURE 3:**
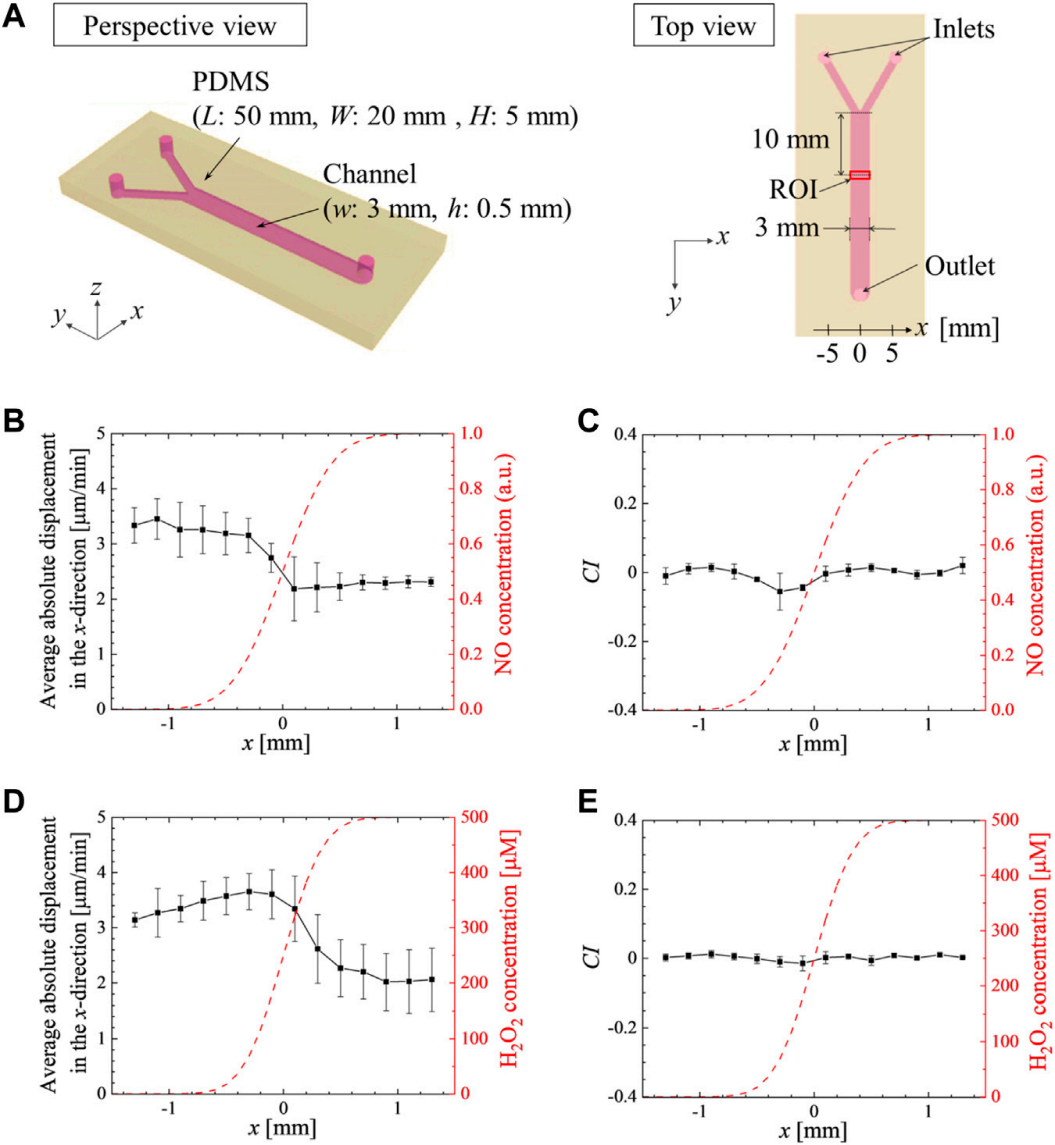
Gradients of SNP or H_2_O_2_ do not induce chemotaxis but reduce the migration speed. **(A)** Schematic of the Y-shaped microchannel device. HL5 culture medium without and with **(B, C)** SNP at 100 μM or **(D, E)** H_2_O_2_ at 500 μM were injected from the left and right inlets, respectively, at 1 mL/h (i.e., the average velocity at 0.37 mm/s in the merged channel). The average absolute values of the x-directional displacements in 1 min **(B, D)**, and the average chemotactic index, *CI =* $\bar{{dl}_{x,i}/{dl}_{i}}$ , **(C, E)** under the chemical gradient plotted as a function of the transverse direction *x*. The error bars show standard deviation of independent experiments (*N* = 3). The calculated profile of chemical concentration *C* is overlapped (red dashed line).

To further exclude a role of ROS and RNS in controlling aerotaxis, wild type cells were placed in a Y-shaped microfluidic device generating a chemical gradient of soluble molecules: either SNP or H_2_O_2_. Higher levels of NO (Figure 3B) or of H_2_O_2_ (Figure 3D) induce a lower motility of *Dictyostelium* cells (negative chemokinesis) but cells did not show specific chemotaxis towards or away from either product, up to 100 µM for SNP and 500 µM for H_2_O_2_ as exemplified by the chemotactic index (Figures 3C, E). These experiments unambiguously indicate that cells do not respond to direct ROS or RNS gradients. As ROS/RNS are not implicated in the observed aerotactic responses, we investigate whether mitochondria are involved in any other way.

### 3.3 Mitochondrial role during aerotaxis

**FIGURE 4:**
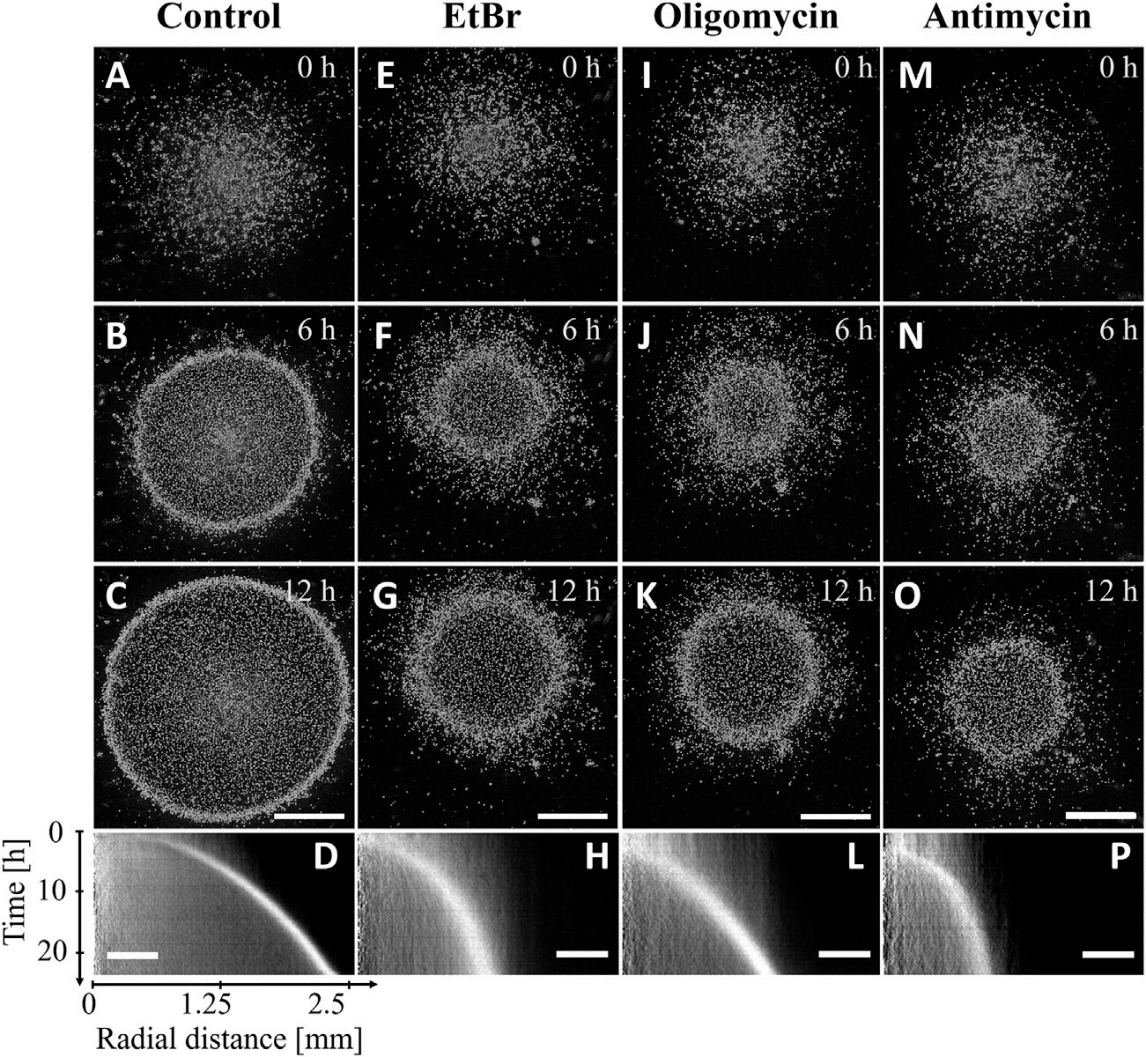
AX2 cells migrate toward oxygen in the confined spot assay even when their mitochondrial activity is impaired. Snapshots **(A,E,I,M)** right after, **(B,F,J,N)** 6 h after and **(C,G,K,O)** 12 h after covering the colony with a glass coverslip non-permeable to O_2_. **(D,H,L,P)** Kymographs of mean image intensity (horizontal axis: radial distance from spot center; vertical axis: time). **(A–D)** AX2 parent cell line (Control), **(E–H)** EtBr treated AX2, **(I–L)** oligomycin treated cells, **(M–P)** antimycin treated cells. Bars: 1 mm and 500 µm for snapshots and kymographs, respectively.

As mitochondria are major oxygen sinks and are potentially involved in direct or indirect oxygen sensing for aerotaxis, three different mitochondrial inhibitors were used on confined wild type cells. Treatment with 10 μg/mL of the mitochondria replication inhibitor EtBr for 5 days was shown to inhibit mitochondrial respiration in *Dictyostelium* cells by 75% and to prevent cell division (Kobilinsky and Beattie, 1977). For our experiments, a pretreatment of 24 h with 30 μg/mL EtBr was used as it results into a strong reduction of cell growth (Chida et al., 2004). This resulted into a reduction of total respiration by 35% comparatively to their untreated control (Supplementary Figure S6D). The confined colony of EtBr-treated cells formed an expanding ring (Figures 4E–H) but with a decreasing migration speed with respect to the non-treated AX2 cells tested (Figures 4A–D; Supplementary Figure S3B) and a slight delay of ring formation time (compared Figures 4D, H). Although it was not measured, cell growth was expected to be strongly reduced since the media contained EtBr during the confinement assays. The EtBr-treated cells were also tested for their aerotactic response in an oxygen gradient (Figures 2D–F). There was no reduction of the aerotactic response and the aerotactic index was similar to untreated cells. Interestingly, on the normoxic side, the addition of EtBr markedly reduced the migration speed of the cells but with no significant reduction on the hypoxic side (Figure 2D).

Oligomycin and antimycin are fast acting inhibitors of mitochondrial respiration that reduce considerably oxygen consumption: by nearly 60% with oligomycin, and 85% with antimycin as measured in this study (Supplementary Figure S6C). As expected, these drugs also reduce considerably cell division (Supplementary Figure S6B). Despite this strong inhibition, an expanding ring of cells was formed in the confined spot assay (Figures 4I–O), but with a 5 h (oligomycin) to 6 h delay (antimycin) as shown with the kymographs of Figures 4D, L, P. In addition, the ring expansion speed was slower than for the untreated AX2 cell colony (control) especially for the antimycin treatment (Figures 4M–P; Supplementary Figure S3B). In the microfluidic assay, oligomycin treated cells (red curves in Figures 2G, H) responded as did the EtBr-treated cells (green curves in Figures 2D, E) with a similar aerotactic displacement along the direction of the gradient but a slightly reduced migration speed on the normoxic side. On the other hand, both migration speed and aerotactic response were reduced by the antimycin treatment (blue curves in Figures 2G, H). Such general reduction of motility is not unexpected as antimycin prevents the cytochrome c oxidase to generate ATP which is required for motility (Wilson et al., 1992).

### 3.4 Modeling of the ring formation and speed of expansion in the different condition tested

**FIGURE 5:**
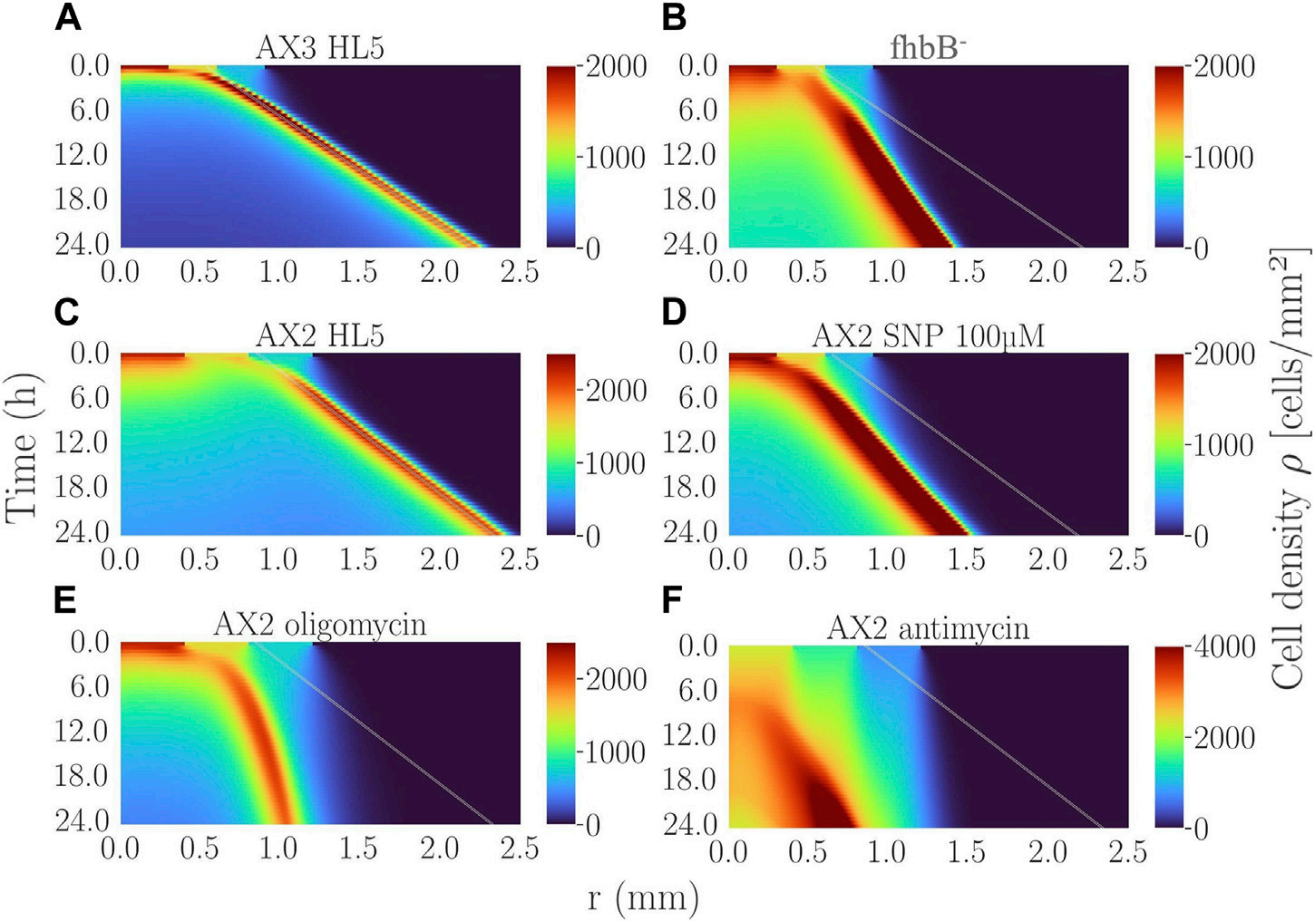
Simulated kymographs of cell density for the various experimental conditions presented in this study: **(A)** AX3 control with 2,000 cells initially plated; **(B)** *fhbB* ^-^ cell line with 2,000 initial cells; **(C)** AX2 control cell line with 5,000 initial cells; **(D)** AX2 cells treated with 100 µM SNP (2,000 initial cells); **(E)** AX2 cells treated with oligomycin (5,000 initial cells); **(F)** AX2 cells treated with antimycin (5,000 initial cells). The line in **(B)** is the slope of the ring-band of **(A)**. The lines in **(E,F)** are the slope of the ring-band of **(C)**. The line in **(D)** comes from the corresponding control AX2 at low initial density not shown. See Supplementary Table S1 for the experimental parameters used.

To interpret experiments, we used the numerical mean-field version of the Go-or-Grow model (Cochet-Escartin et al., 2021). Briefly, the rapid oxygen consumption by cells in the confined area and the very selective aerotactic cell response are the two key ingredients for ring formation and propagation. Ring formation may occur even in the absence of proliferation (Biondo et al., 2021; Cochet-Escartin et al., 2021). However, proliferation is necessary to sustain a stable long-lived ring (Rieu et al., 2022). The ring speed depends mostly on the aerotactic speed and slightly on the proliferation rate. Here, we simulated the experiments with various O_2_ diffusion constants values taken from literature and found that the changes in the ring speed between the various extreme literature values were negligible (Supplementary Figure S7 and its caption). The ring speed also did not depend on initial cell density, only the formation time depended on it: the denser, the quicker the ring formation and the more it appeared in the peripheral position of the initial spot (Supplementary Figure S8). After adjusting the aerotactic response from microfluidic experiments, and the initial cell density profile and other parameters from experiments, the model predicted a ring formation within an hour for AX2 and AX3 cell spots (Figures 5C, A) in agreement with experiments (Figures 4D, 1D). The simulated ring propagated at constant speeds of ∼1.1 and 1.2 μm/min for AX2 and AX3, respectively. These values were in good agreement with the range of measured ring speeds (Supplementary Figures S3A–C), given the experimental variability.

All other cell lines tested in this study presented an aerotactic response (i.e., $v_{x}>0$ ) in the region of 0%–2% O_2_ (Figures 2B, E, H). Simulated rings of *fhbB* ^-^ mutant (Figure 5B) were 50% slower and much wider than those of the parent cell line AX3 (Figure 5A) as the *x*-directional velocity *v*_*x*_ was only 37% of their parent one. But, the rings appeared as quickly as their parent did as the cellular oxygen consumption or proliferation was not impaired. The rings of AX2 treated with 100 µM SNP (Figure 5D) were 21% slower than those of non-treated cell line (Figure 5C) as the *x*-directional velocity *v*_*x*_ was also reduced by 21% with respect to their parent. They were also wider but not significantly delayed as the oxygen consumption of this condition was only slightly decreased with respect to the control. These findings were in very good agreement with experimental observations (Figures 1D, L; Supplementary Figures S5D, H).

Oligomycin and antimycin treatments corresponded to a very different set of parameters. The aerotactic directional migration speed *v*_*x*_ of oligomycin-treated cells was similar to the non-treated AX2 (Figure 2H) but they did not proliferate at all (Supplementary Figure S6B). As a result, once initiated the ring expanded poorly (Figure 5E) and even stopped after a while. The ultra-low oxygen consumption of antimycin-treated cells made the ring appear at very late simulation times (about 7 h in Figure 5F) comparable to experimental observations (Figure 4P).

One can conclude from these simulations that.

(i) Rings formed only in presence of aerotaxis.
(ii) Ring speed mostly correlated with the aerotactic strength (*x*-directional velocity proportional to aerotactic bias).
(iii) Ring formation time did not correlate with the aerotactic strength but with the overall oxygen consumption by the cells in the confined area.

## 4 Discussion

*Dictyostelium* motility towards extracellular cAMP has been described extensively and served as a model for chemotaxis in other organisms (Pal et al., 2019). cAMP acts through G protein-coupled receptors (GPCR) to regulate actin polymerization and cell motility. Spatial gradients of cAMP between the front and the back of the cells are detected and amplified intracellularly through second messengers to direct motility. *Dictyostelium* uses similar signaling mechanisms to respond to a wide variety of signals such as temperature (Poff & Skokut, 1977), light (Fisher, 1997; Marée et al., 1999), electric current (Zhao et al., 2002; Sato et al., 2007), and chemical substances such as folate (Pan et al., 2016). In all these characterized situations, cells perceive differences of the signal levels on the plasma membrane, usually using a receptor, and integrate them to become polarized and direct their motility (Devreotes & Horwitz, 2015). In the present case, the initial signal is oxygen, a molecule normally present in high amount in the environment and constantly used for basic metabolism. There is no known cell surface receptor for oxygen and its hydrophobic properties allow its free diffusion through membranes before being metabolized. Inactivation of the G-beta protein required for transducing most GPCR signal does not prevent aerotaxis (Biondo et al., 2021).

To identify the signaling mechanism, we tested mutants in gene-coding potential oxygen-sensing proteins using a confined spot assay to observe an aerotactic ring, and then further dissected this phenomenon to model and distinguish what aspect of the cell properties was affected by the mutation. The same strategy was then used when challenging cells with various chemicals. Among tested mutants, the *fhbB* ^*-*^ and *fhbA* ^*-*^ *B* ^*-*^ strains were the one presenting a somewhat altered ring formation with the micro-colony confining test. However, the fact that a ring was formed and expanded, even if irregular and at lower speed, indicates that the cells still present some aerotaxis, which was confirmed when imposing an oxygen gradient. The strong reduction but not complete abolition of the aerotactic index (*AI*_max_, Figure 2C) could indicate a direct role for flavohemoglobins in sensing oxygen gradients, but can also be an undirect effect that mask the cell aerotaxis. Our further assays with imposed oxygen or chemical gradients and simulation argue for an undirect role of flavohemoglobins and NO. *Dictyostelium* cells did not move towards or away from NO generated by SNP in the microfluidic RNS gradient experiment (Figure 3), excluding a role for NO to act as secondary gradient to orient cell motility. If NO was used as a signal to orient cell or even to fine tune aerotaxis, swamping the cells with 500 µM SNP should reduce their aerotaxis, which was not the case. Instead, SNP impairs general motility by reducing the average cell speed, this effect being more pronounced in hypoxic conditions (Supplementary Figure S3D). As SNP reduces more cell motility in hypoxic condition and flavohemoglobins expression is induced at low oxygen (Table 1), it suggests that those enzymes main function is to protect cells from toxic effects of NO generated upon hypoxia. The weak aerotactic response of the *fhbB* ^-^ cells cannot be fully explained by the motility reduction caused by acute exposure to NO in hypoxic condition. It seems that the strains present other defects, maybe due to adaptation to chronic exposure to self-generated NO. In most microorganisms, low level NO is produced as part of nitrogen metabolism, and flavohemoglobins regulate NO homeostasis (Cánovas et al., 2016). It is thus likely that inactivation of *fhbB* results into elevated levels of NO even during normal growth that would cause the mutants’ phenotypes. The observation that *fhbB* ^-^ cells were bigger than the wild type without being polynucleated and more sensitive to toxicity when exposed to high glucose concentration suggests a broad metabolic unbalance (Iijima et al., 2000). Differences between short-term and long-term exposure to NO have been observed when incubating *Dictysotelium* with SNP over prolonged period (Taminato et al., 2002). Wild type cells’ growth is little affected during the first 20 h but protein kinase A (PKA) becomes transiently activated after 12 h of SNP treatment through the yakA pathway as part of a stress response that then leads to reduced cell growth. The protein kinase, yakA, regulates cell division, actin filament polymerization, transition from growth to development and the arrest of growth in response to stresses, including exposure to NO (Mantzouranis et al., 2010). The *fhbB* ^-^ cells might thus present a low-level activation of the yakA pathway in response to a constant stress due to elevated exposure to RNS, leading to abnormal cell size and motility. Thus, the *fhbB* ^-^ mutant appears to be affected in aspects unrelated to the rapid adaptation to hypoxia that characterize aerotaxis. As such, the data obtained with SNP treatment appear more significant than the one with the *fhbB* ^-^ strain and lead to the conclusion that aerotaxis is not directly controlled by NO signaling.

Based on our experimental data with various strains and conditions, we used a model allowing us to explain many outcomes of ring formation and propagation in our confined spot assay. Cells first need to generate an oxygen gradient through respiration. Changes in cell number or respiration capability will mostly affect the timing to generate a sufficiently steep gradient for cells to detect. But eventually, aerotactic motility occurs and a ring is formed as shown with the various mitochondrial inhibitors that reduced but not fully abolished oxygen consumption. Surprisingly, the inhibition of mitochondrial respiration did not block aerotactic ring formation in *Dictyostelium* contrary to epithelial or breast tumor cells (Deygas et al., 2018; Mikaelian et al., 2022). The inhibition of mitochondrial respiration by oligomycin or antimycin in human cell lines likely prevents the formation of the steep oxygen gradient needed to direct the outward cell motility (Mikaelian et al., 2022). Like many protists, *Dictyostelium* possesses an alternate oxidase that consumes oxygen and is not blocked by cyanides (Kimura et al., 2010), explaining how the cells retain a significant respiration capability even in presence of antimycin or oligomycin. With *Dictyostelium*, our results allow to distinguish the role of mitochondria as a sink for oxygen to generate a gradient from its putative functions as oxygen sensor. As mitochondria are not necessary for aerotaxis, it excludes their direct role as oxygen sensors for *Dictyostelium*. Mikaelian et al. have also shown that mitochondria are dispensable for oxygen sensing in human cancer cells by showing that cells with impaired mitochondria will follow the gradient generated by normal one. Since the main ATP biosynthesis pathway is dispensable, a control of aerotaxis through a secondary signal based on energy depletion, such as ADP/ATP ratio is also unlikely. Antimycin not only blocks ATP synthesis but also induces ROS production in *Dictyostelium*’s mitochondria (Downs et al., 2021), yet aerotaxis remained normal. Aerotaxis was also unaffected upon treatment with H_2_O_2_ or Tiron, a ROS scavenger. Taken together, these data also exclude a role for ROS as secondary signal for aerotaxis in *Dictyostelium*. Controversially, Biondo et al. suggested that *Dictyostelium* cells utilized intracellular H_2_O_2_ as an activator or an enhancer of aerotactic migration (Biondo et al., 2021), similar to mammalian cells (Deygas et al., 2018). They performed a confined spot assay with a catalase-deficient strain and reported that its aerotactic ring formation was much quicker than the wild type. However, our experiments and modeling of aerotactic ring shows that the timing of its formation is absolutely not dependent upon the strength of aerotaxis, but is determined by the number of cells in the micro-colony and their oxygen consumption. The presented data illustrate well these concepts: the mitochondrial inhibitors that reduce oxygen consumption without influencing aerotaxis do delay the timing of the ring formation, while the *fhbB* ^-^ mutant with reduced aerotaxis but normal respiration form a ring at the same time than the wild type. In mammalian cells, there are clear evidence that aerotactic motility requires the modification of the receptor tyrosine kinase EGFR by oxidative stress (Deygas et al., 2018; Mikaelian et al., 2022). As the *Dictyostelium* genome is devoid of any receptor tyrosine kinase (Eichinger et al., 2005), it is not surprising that this specific mechanism is not conserved. As some of the strains tested in Mikaelian et al. (2022) do not express EGFR, alternate oxygen sensors might also exist in human. While the reported results did not allow to identify the molecular mechanism behind *Dictyostelium* aerotaxis, we were able to exclude the most expected contributors: mitochondria or secondary signals linked to ROS and RNS.

## Data Availability

The original contributions presented in the study are included in the article/Supplementary Material, further inquiries can be directed to the corresponding authors.
