# Supplementary material for: The aerotaxis of Dictyostelium discoideum is independent of mitochondria, nitric oxide and oxidative stress
Source: Front Cell Dev Biol. 2023 Jun 15;11:1134011. doi: 10.3389/fcell.2023.1134011 (PMC10307954; doi:10.3389/fcell.2023.1134011)
Supplement: Supplementary file 1 [file Presentation1.pdf]

Supplementary Material

The aerotaxis of *Dictyostelium discoideum* is independent of mitochondria, nitric oxide and oxidative stress

Satomi Hirose[1,2,3*,$], Julie Hesnard[4,$], Nasser Ghazi[4], Damien Roussel[5], Yann Voituron[5], Oliver Cochet-Escartin[4], Jean-Paul Rieu[4*], Christophe Anjard[*4], Kenichi Funamoto[1,2*]

*** Correspondence:**

Satomi Hirose: satomih@mit.edu,

Jean-Paul Rieu: jean-paul.rieu@univ-lyon1.fr

Christophe Anjard: christophe.anjard@univ-lyon1.fr

Kenichi Funamoto: funamoto@tohoku.ac.jp

1 Supplementary Data

1.1 Supplementary Table

Supplementary Table S1 : Experimental parameters used for simulations using the Go-or-Grow model (Fig. 5). With oligomycin, we used a no proliferation condition.

Cell line (panel index of Fig. 5)	AX3 (A)	fhbB- (B)	AX2 (C)	AX2 (D)	AX2 (E)	AX2 (F)
Treatment	None	None	None	SNP 100 µM	Oligomycin 10 µg/ml	Antimycin 37.5 µM
Doubling time T = ln(2)/r_0 (h)	10.65	6.81	7.34	8.69	∞	17.57
Consumption b_0 (10^{-17} mole/cell/s)	5.47	5.32	4.73	3.43	1.98	0.80
Aerotactic *x*-directional speed v_x (µm/min)	1.11	0.23	1.00	0.49	0.90	0.40
Diffusion constant (µm^2/min)	30.00	14.70	30.00	30.00	24.94	6.64

1.2 Supplementary Figures

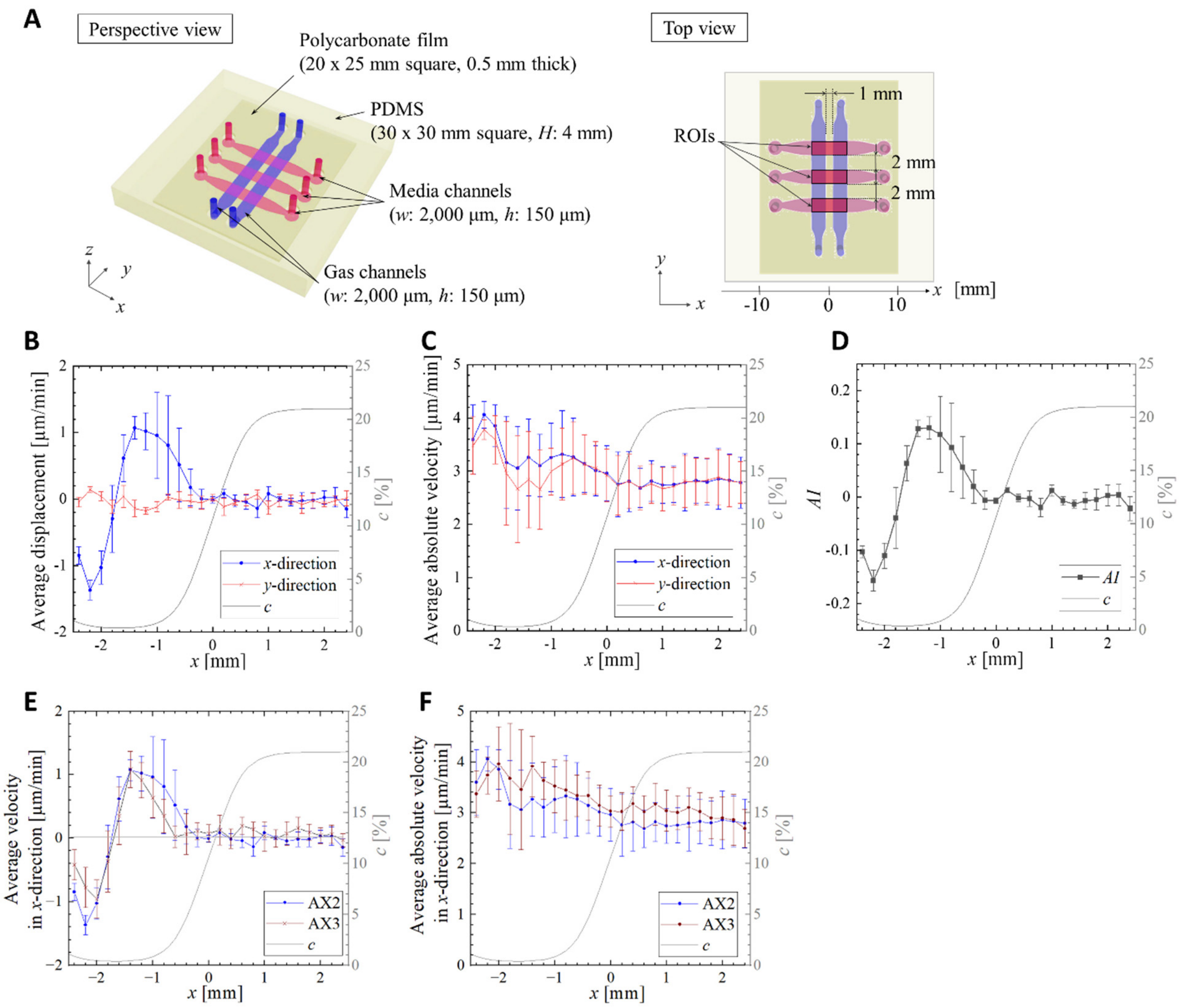

Supplementary Figure S1: (A) Microfluidic double-layer device to probe the aerotactic response of cells. (B-D) Average displacement, average absolute velocity speeds, and aerotactic index *AI* of AX2 cells in HL5 (control) (Hirose et al, 2022). (E-F) The comparison of average velocity and average absolute velocity in *x*-direction between two standard strains, AX2 and AX3. They showed almost the same tendency on both motility and aerotaxis. The error bars show standard deviation of independent experiments ($N = 4$ for AX2, $N = 6$ for AX3).

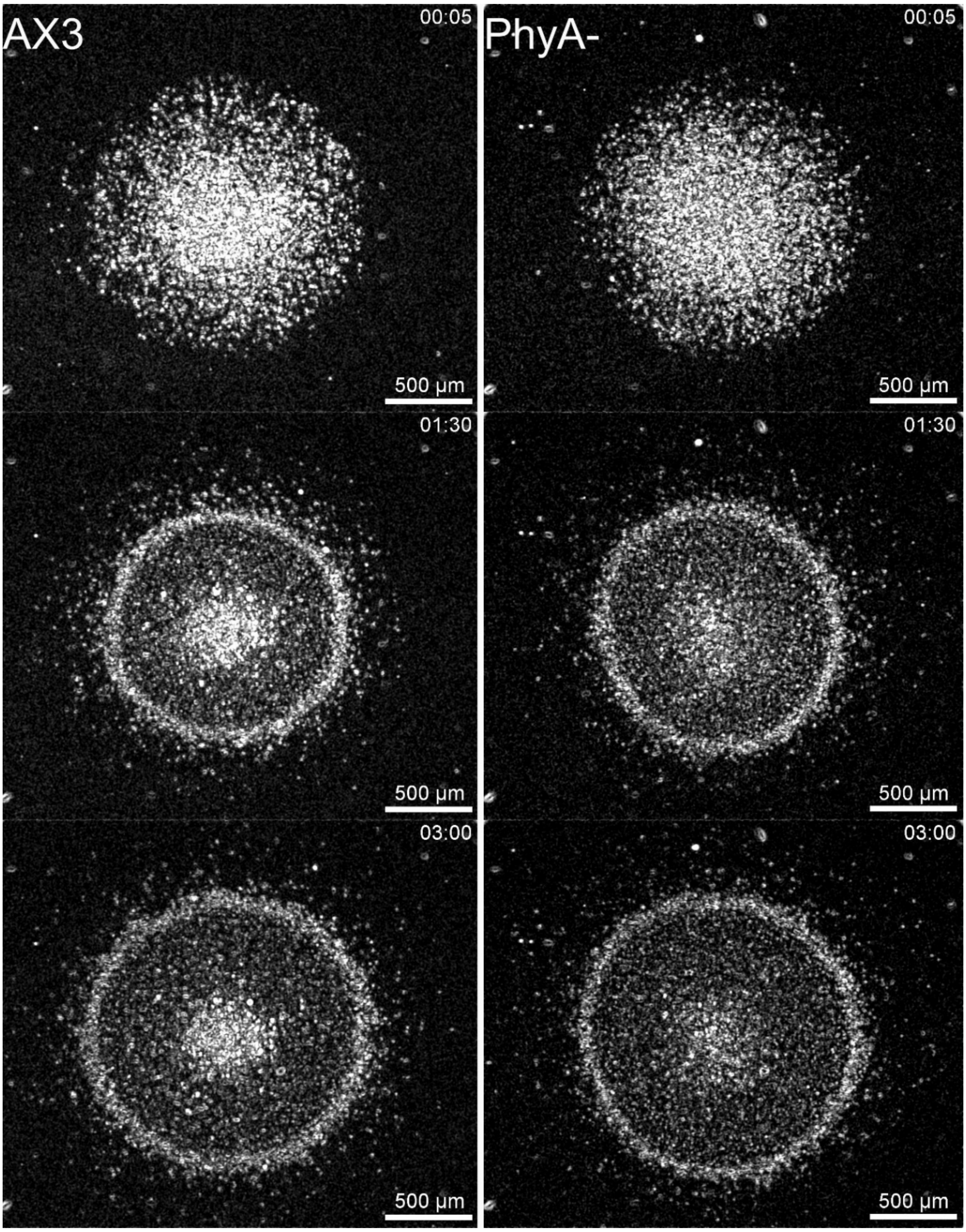

Supplementary Figure S2: PhyA- cell line (right column) presents a normal phenotype in the confined spot assay with respect to its parent AX3 (left column): a ring of cells quickly arises from the center of the colony and propagates outwardly. Time in h:min from the initial confinement time is indicated on the top right of each panel. Ring speeds calculated at early times (30 min-3 h) are 2.8 ± 0.5 µm/min and 2.6 ± 0.2 µm/min for the PhyA- and AX3 cell lines respectively (mean ± SD, $N = 6$). These values are similar to the one measured at early times in Fig. 1D.

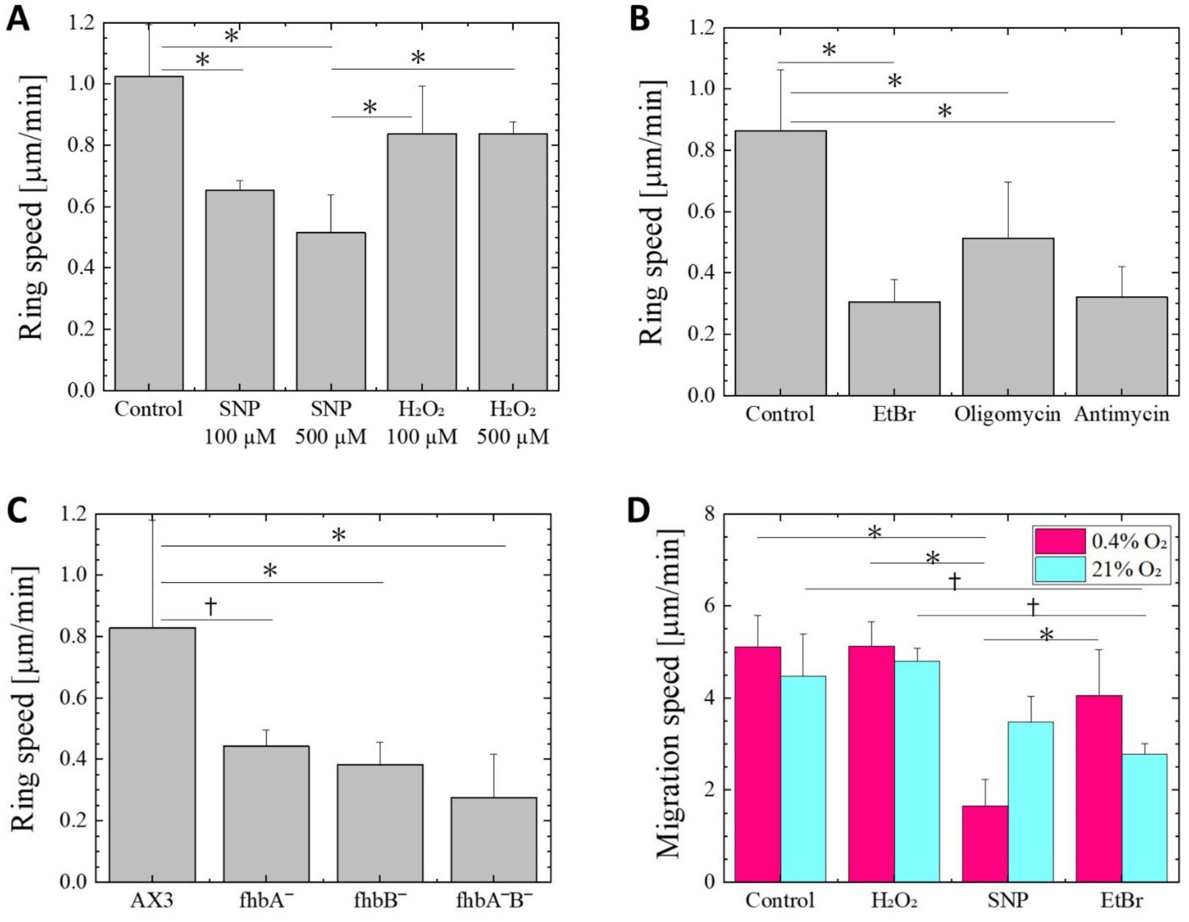

Supplementary Figure S3: (A-C) Ring speed measured at long times (~12 h-24 h) in the spot assay for the various strains and for various treatments described throughout the manuscript: Controls are the AX2 cell lines in (A) and (B). (D) Mean displacement speed measured in the hypoxic and normoxic sides of the cell channel of the microfluidic device under a 0-21% gradient for the AX2 cell line submitted to various treatments (SNP at 100 µM, H_2O_2 at 100 µM, and EtBr at 10 mM). The error bars show standard deviation of independent experiments ($N \geq 3$). Significant differences were assessed by one-way or two-way ANOVA followed by Tukey's post hoc test for multiple comparisons. †$p < 0.1$; *$p < 0.05$.

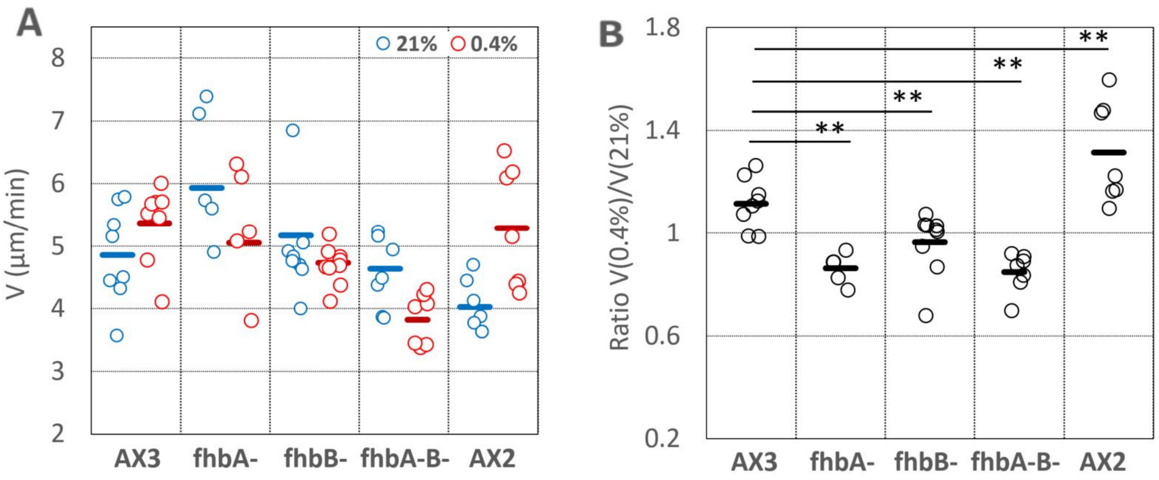

Supplementary Figure S4: (A) Instantaneous speed of various cell lines in isotropic normoxic (21% O_2) and hypoxic (0.4% O_2) conditions and (B) ratio of speed between hypoxic and normoxic conditions. Cells were plated in 6-well plates with a polystyrene bottom at a low density (~3000 cell/cm^2) and were submerged with 1.5 mm HL5 medium. The plates were inserted in a homemade environmental chamber for gas exchange. The three flavohemoglobin KO, the AX2 and AX3 cell strains were simultaneously imaged with an inverted microscope equipped with a motorized stage. Cell trajectories were tracked at 21% O_2 for 90 min and the assay was repeated at 0.4% O_2 for another 90 min after a 90 min equilibration period to exchange O_2 from the surrounding gaseous phase to the culture medium. Absolute values of the instantaneous speed V were computed from displacement at 45 s-time intervals. Each condition (blue and red circles for 21% and 0.4%, O_2 respectively) was repeated 5 to 8 times. Thick horizontal bars represent mean values for each condition. Significant differences were assessed by one-way ANOVA followed by Tukey's post hoc test for multiple comparisons. $^{**}p < 0.01$. We did compare only pairs between the AX3 strain and another strain.

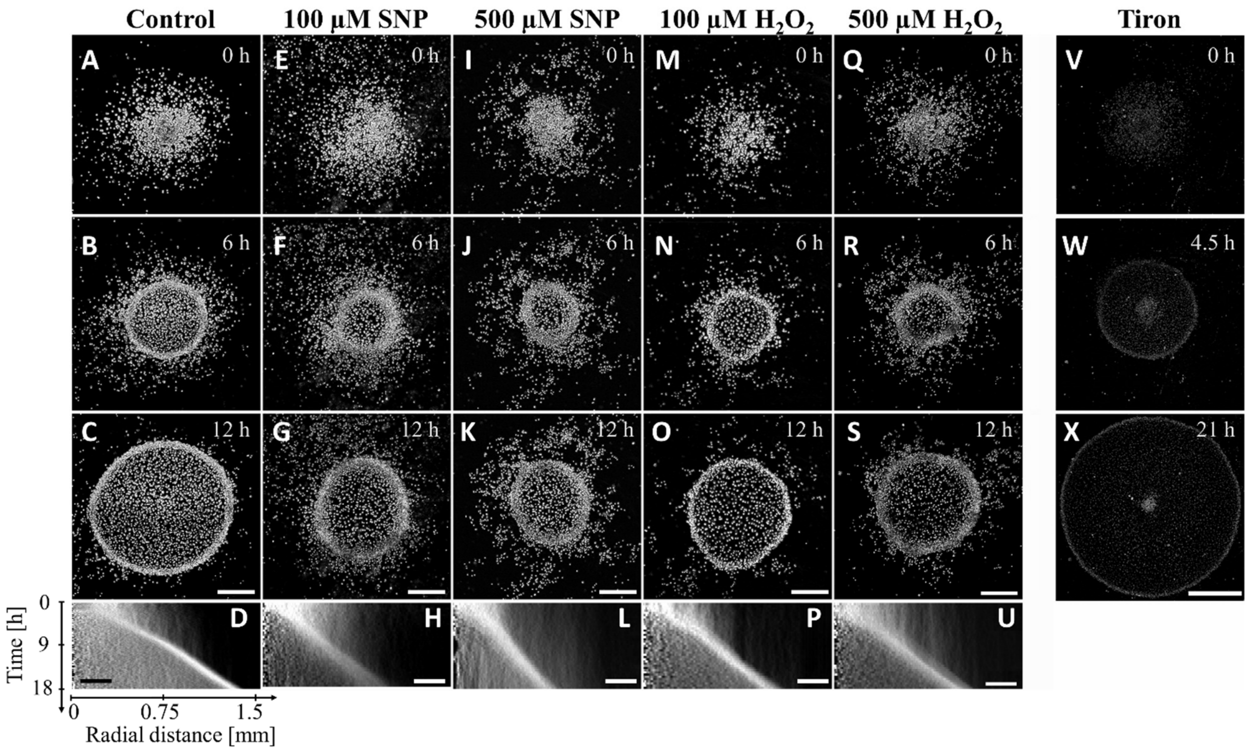

Supplementary Figure S5: ROS do not qualitatively affect ring initiation nor ring long term propagation. Snapshots right after (A, E, I, M, Q, V), 4.5 h after (W), 6 h after (B, F, J, N, R), 12 h after (C, G, K, O, S) and 21 h after (X) covering the colony with a cover glass non permeable to O_2: cells quickly consume available O_2 and move outwardly with a characteristic ring front. (D, H, L, P, U) Kymographs of mean image intensity (horizontal axis, radial distance from spot center, vertical axis time). (A-D) AX2 parent cell line, (E-H) AX2 treated with 100 µM SNP, (I-L) AX2 treated with 500 µM SNP, (M-P) AX2 treated with 100 µM H_2O_2, (Q-U) AX2 treated with 500 µM H_2O_2, (V-X) AX2 treated with 10 mM Tiron. Snapshot bars: 500 µm except (V-X) 1mm; kymograph horizontal bars: 250 µm.

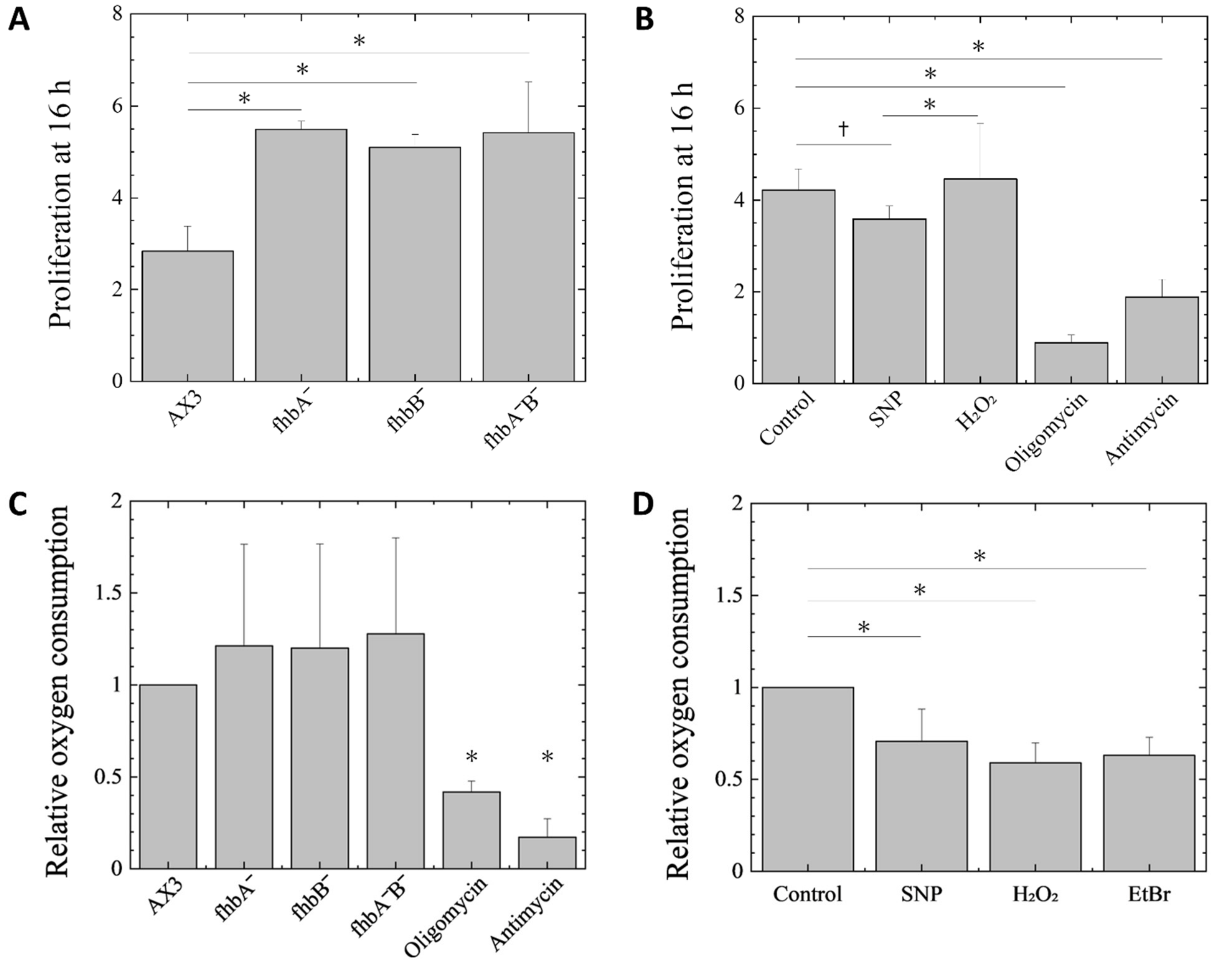

Supplementary Figure S6: (A, B) Proliferation at 16 h (fold increase in the number of cells between 0 and 16 h) for the various strains and conditions indicated under the bars. (C, D) Ratio of oxygen consumption for the various strains and for the various treatments described throughout the manuscript with respect to their control. Control for (B) is the AX2 cell line. Control for (C) is the AX3 cell line which consumes 5.5×10^{-17} moles/cell/s. Control for (D) is the AX2 cell line. The basal consumption value of the control depends on the experimental series: $5.3 \pm 2.4\times10^{-17}$ moles/cell/s for the SNP/H_2O_2 experimental series (mean ± SD, $N = 3$), and $9.6 \pm 2.1\times10^{-17}$ moles/cell/s for the EtBr series ($N = 4$), while the slightly smaller consumption value of AX2 cell line indicated in SI Table S1 was measured over much more experiments ($N = 15$). In (A-D), the SNP concentration is 100 µM, the H_2O_2 concentration is 100 µM, the Oligomycin concentration is 10 µg/ml, the Antimycin concentration is 37.5 µM. †$p < 0.1$; *$p < 0.05$.

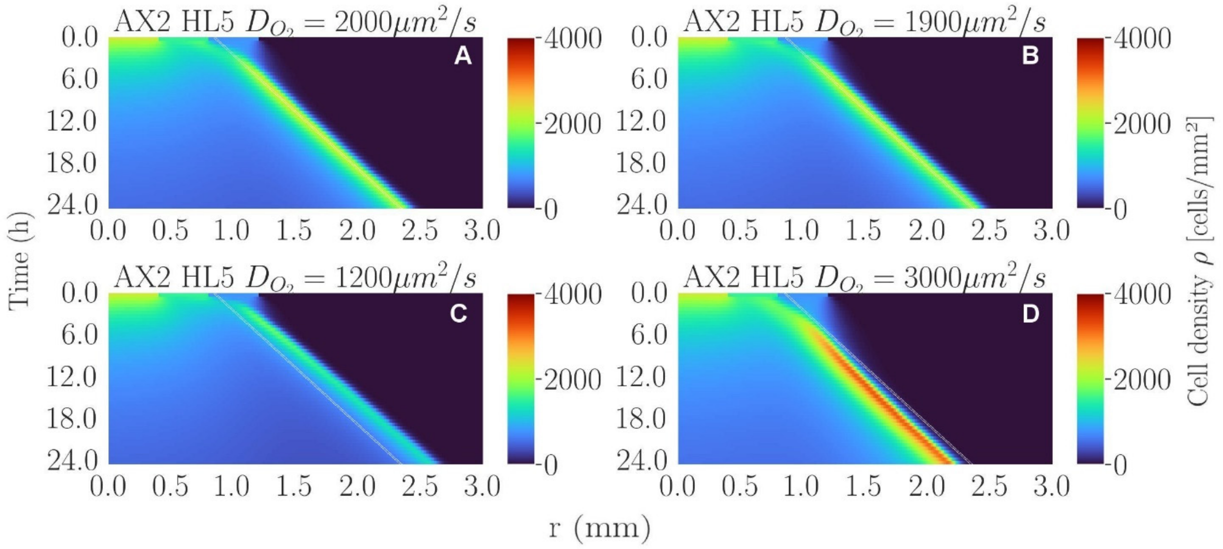

Supplementary Figure S7: Simulated kymographs of cell density with 5000 initial cells for AX2 cells (see Fig. 5(C) as control) with various O_2 diffusion constants D_{O2} taken from literature. Although we did not measure the diffusion constant value D_{O2} in HL5 medium at 22°C, we can assume that it is close to 2000 µm²/s, the pure water value at 20°C: on one hand, salt and sugar present in HL5 decrease D_{O2} by about 100 µm²/s, but on the other hand temperature increases D_{O2} by about 50 µm²/s/°C (Goldstick et al, 1976). (A) D_{O2} = 2000 µm²/s; (B) D_{O2} = 1900 µm²/s, roughly corresponding to D_{O2} in pure water with 2 g/L salt and 10 g/L sugar at 20°C (Jamnongwong et al, 2010), the respective salt and sugar concentrations in our HL5 medium; (C) D_{O2} = 1200 µm²/s, corresponding to D_{O2} in plasma at 25°C (Goldstick et al, 1976); (D) D_{O2} = 3000 µm²/s, corresponding to D_{O2} in pure water at 37°C (Goldstick et al, 1976). The lines in (A-D) correspond to the slope of the ring-band in (A). Large variations in D_{O2} modify slightly the ring density and the radial position of the band which is more outside when D_{O2} is decreased (C) with respect to (A). The ring speeds were not greatly modified by D_{O2} as we measured 1.071 µm/min, 1.071 µm/min, 1.128 µm/min, and 1.027 µm/min at t = 18 h for the O_2 diffusion constants values of (A-D), respectively.

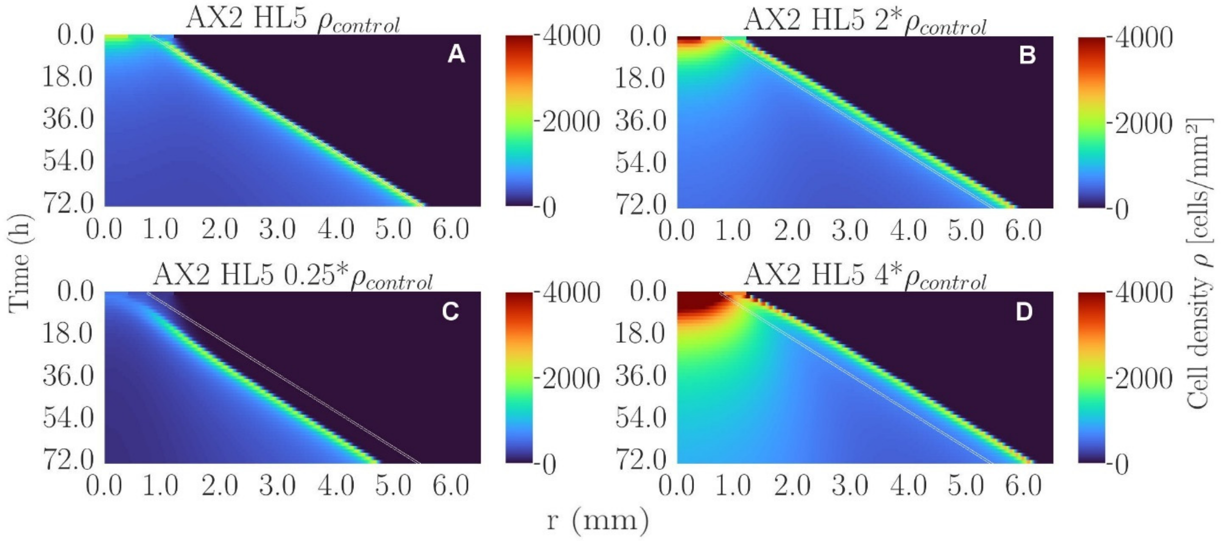

Supplementary Figure S8: Simulated kymographs of cell density over 72 h for AX2 cells (see Fig. 5(C) as control) with various initial densities ρ in a spot of 1.2 mm diameter: (A) $\rho = 5\times10^3$ cells/spot, (B) $\rho = 1\times10^4$ cells/spot, (C) $\rho = 1.25\times10^3$ cells/spot, and (D) $\rho = 2\times10^4$ cells/spot. The lines in (A-D) correspond to the slope of the ring-band in (A). A lower density delays ring formation time and commences further inside the initial spot area: compare (A) and (C). On the other hand, a higher density causes the ring to appear immediately and especially on the periphery of the zone of the initial spot: compare (A) and (D). However, the propagation speeds are only very marginally modified at 1.143 µm/min, 1.151 µm/min, 1.122 µm/min, and 1.158 µm/min for the ring-bands in (A-D), respectively.

1.3 References

Goldstick, T. K., Ciuryla, V. T. & Zuckerman, L. (1976) Diffusion of oxygen in plasma and blood. *Oxygen Transport to Tissue—II*, 183-190.

Hirose, S., Rieu, J.-P., Cochet-Escartin, O., Anjard, C. & Funamoto, K. (2022) The Oxygen Gradient in Hypoxic Conditions Enhances and Guides *Dictyostelium discoideum* Migration. *Processes*, 10(2), 318.

Jamnongwong, M., Loubiere, K., Dietrich, N. & Hébrard, G. (2010) Experimental study of oxygen diffusion coefficients in clean water containing salt, glucose or surfactant: Consequences on the liquid-side mass transfer coefficients. *Chemical Engineering Journal*, 165(3), 758-768.
